# Mapping evidence on the distribution of paediatric cancers in sub-Saharan Africa: a scoping review protocol

**DOI:** 10.1186/s13643-019-1184-4

**Published:** 2019-11-07

**Authors:** Sehlisiwe R. Ndlovu, Desmond Kuupiel, Themba G. Ginindza

**Affiliations:** Discipline of Public Health Medicine, School of Nursing and Public Health, University of KwaZulu-Natal, Durban, South Africa

**Keywords:** Paediatric cancers, Prevalence, Mortality, Incidence, Morbidity, Risk factors, Trends, HIV

## Abstract

**Background:**

Paediatric cancers account for a minor fraction of deaths and hence receive little attention from policymakers. In low-income countries, the absence of comprehensive national paediatric strategies results in a lack of access for a majority of children with cancer. In sub-Saharan Africa (SSA), the burden of childhood cancers is underestimated due to a lack of paediatric cancer registries, poor health care systems and competing healthcare needs. The objective of this study is to map evidence on the distribution of paediatric cancers in the SSA region.

**Method:**

A scoping review will be conducted to map literature on the distribution of paediatric cancers in SSA**.** An electronic literature search will be conducted from the following databases: PubMed, Google Scholar, EBSCOhost (CINAHL and Health Source) and World Health Organization (WHO)/International Agency for Research in Cancer (IARC) (GLOBOCAN databases). We will also search the reference lists of included studies to source relevant literature. A pilot search was conducted to determine the feasibility of the study. Study selection will be guided by the inclusion and exclusion criteria. After charting the data, a descriptive overview of the studies will be presented in a narrative format. An account of the study characteristics will be described in this narrative. The analysis will be mainly based on mapping the country-specific outcomes emerging from the studies, and a numerical summary of these outcomes will be conducted. Tables, maps and charts will be produced and presented in the result section.

**Discussion:**

This review study will identify existing research gaps for future research to influence policy implementation and to improve the availability of diagnosis and treatment of paediatric cancers in SSA.

## Background

Globally, cancer remains the leading cause of disease-related death in children [1]. The estimated 5-year prevalence of cancers in 2018 for children 14 years and below is 606,982 [2]. Children represent 41% of the total population residing in Africa, and in some of these regions, children constitute more than half of the population [1]. In 2018, the estimated number of prevalent paediatric cancers is 103,016 [2]. The most prevalent cancers in children 14 years and below are leukaemia (19,246), kidney cancer (14,002), non-Hodgkin lymphoma (13, 368), brain and nervous system (6317) and Kaposi sarcoma (3101) [2]. The prevalence rates in Africa may be underestimated due to a lack of proper diagnosis and childhood cancer registration [3, 4]. The actual distribution of these malignancies in SSA is therefore not truly reflected because of poor health care systems and competing health care needs, making it difficult to develop paediatric cancer control programmes [5].

Paediatric cancers receive little attention from local policymakers and global health agencies due to the fact that it accounts for a minor fraction of deaths ranging from 0.5% in high-income countries (HIC) to approximately 5% in low-income countries [6]. The absence of comprehensive national paediatric strategies results in a lack of access to care for a majority of children with cancer in low-middle-income countries (LMICs) [7]. In an effort to improve the survival rates of children affected by cancer, the International Agency for Research in Cancer (IARC) made a collaboration with cancer registries and partners around the world [6]. They work closely to generate and provide global data which is important in developing and monitoring efficient strategies for the early detection and care for paediatric cancer [4, 6]. As all registries must comply with standard criteria, this international collaboration will bring about an improvement in local data and information. However, this poses a challenge in SSA as there is limited information on the distribution of various childhood malignancies in Africa, and the policy is based on information from other countries [1].

Infection with HIV increases the risk of cancer [8]. In HICs, it is estimated that 2.5% of children with HIV will develop cancer [9]. More than two-thirds of people living with HIV reside in SSA [9] and life expectancy has increased due to the introduction of highly active antiretroviral treatment (HAART). Most studies linking cancer to HIV infection are based on the adult population [10]. To provide a better understanding of the spectrum of HIV-associated cancers in the paediatric population and subsequently come up with feasible and effective strategies for their diagnosis and cure, there is a need for well-planned population-based studies [9]. The starting point for effective analytical epidemiological studies will be understanding the trends of these malignancies prior and in the HIV era.

Therefore, this scoping review aims to map evidence on the distribution of paediatric cancers in sub-Saharan Africa. Understanding the distribution of paediatric cancers will help provide the basis for the design of sound clinical and public health programmes. It will provide a visual insight of the extent and magnitude of the prevalence, incidence and mortality rates of paediatric cancers in the SSA region. It also provides the basis for the design of research programmes in individual countries that will support the health care effort required to prevent, cure and subsequently increase the survival rates in paediatric cancer patients SSA.

## Methodology

### Scoping review

The proposed scoping review will be conducted based on the existing methodological framework [11]. The framework stipulates the following steps: identifying the research question, identifying relevant studies, study selection, charting the data and finally collating, summarising and reporting the results.

### Identifying the research question

The main research question for this scoping study is what are the types of evidence and concepts behind estimating the distribution of paediatric cancers in Sub-Saharan Africa?

The sub-research questions are what are the risk factors associated with paediatric cancers in SSA and what are the trends in paediatric cancer distribution in relation to HIV in SSA?

The study will use an amended PEO (Population, Exposure and Outcome) framework to determine the eligibility of the research question (Table 1).

**Table 1:** PEO framework for eligibility of research question

Criteria	Determinants
Population	Children with cancer in sub-Saharan Africa
Exposure	Paediatric cancers
Outcome	Mortality, incidence and prevalence estimations showing evidence on the distribution of paediatric cancers in SSA
	Risk factors
	Interplay between trends in paediatric cancers and HIV

### Identifying relevant studies

The studies will be identified by searching literature published in English with no date limit. An electronic literature search will be conducted from the following databases: PubMed, Google Scholar, EBSCOhost (CINAHL and Health Source) and World Health Organization (GLOBOCAN databases). We will also search reference lists of included studies to source relevant literature. A detailed search record will be documented and will include the following: date of search, database, keywords and number of studies.

The following keywords will be used in the search: “children”, “paediatric”, “childhood”, “cancer”, “paediatric cancers”, “childhood cancers”, “risk factors”, “prevalence”, “incidence”, “morbidity”, “mortality”, “HIV”, “trends” and “Sub-Saharan African country”. Medical Subject Headings (MeSH) terms will be used in the search. Boolean terms, AND/OR, will be used to separate the keywords. We have conducted a pilot search using the above keywords to determine the feasibility of this study (Appendix 1).

### Study selection

The eligibility criteria of this study will be as follows:

#### Inclusion criteria

The inclusion criteria are primary studies on paediatric cancers, studies reporting evidence of paediatric cancer in SSA, quantitative studies, studies reporting relevant outcomes of this study, studies published in all languages and studies reporting cancers in children in the 0–14 years age range.

#### Exclusion criteria

We will exclude studies reporting on cancers in children who are 15 years and above, studies reporting evidence on population outside SSA and qualitative studies.

**Fig. 1:**
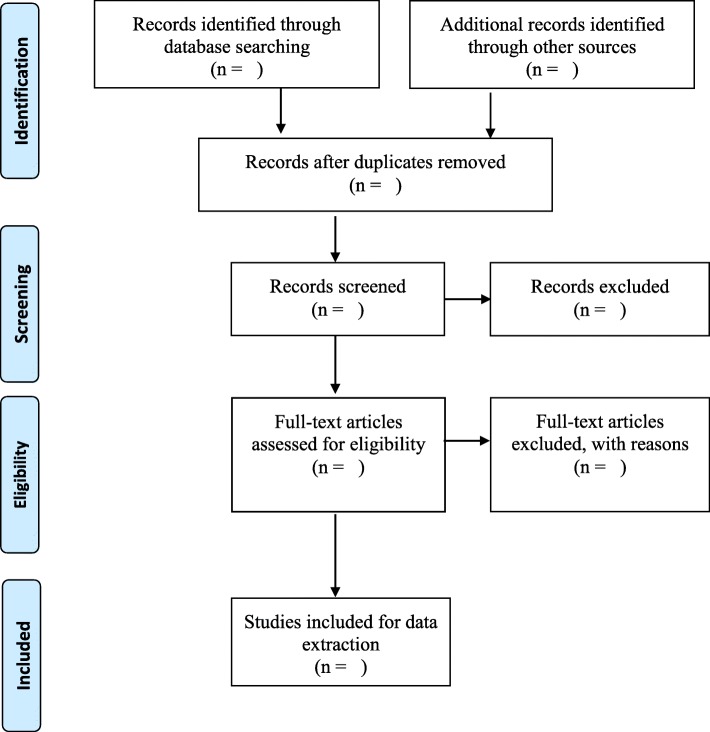
PRISMA flow diagram

A comprehensive search and screening of the study titles will be performed by one reviewer from the databases. After the identification of studies with relevant titles, the studies will be exported to Endnote X8 software. Following this, duplicates will be removed and the library shared amongst the reviewers. Two independent reviewers will conduct both abstract and full-article screening guided by the inclusion and exclusion criteria. In the event where a study/article could not be retrieved from a database, we will request for assistance from the University of KwaZulu-Natal library services or contact the authors to request for the full article. In the case of discrepancies between the reviewers at the abstract screening stage, consensus will be reached through discussion between the reviewers. However, a third reviewer will be engaged to resolve disagreement at the full-article stage. Screening results will be reported using the adapted Preferred Reporting Items for Systematic Reviews and Meta-Analyses (PRISMA) guidelines [12], as shown in Fig. 1.

### Charting the data

Data will be extracted using an electronic data extraction tool as shown in Table 2. The following information will be extracted: author and year of publication, study title, study aim, study design, population characteristic, gender, country, summary of finding and outcome measures employed. Outcome measures to be analysed will include measures of disease distribution, incidence, prevalence and mortality. Time trends will also be analysed from the included studies to ascertain if there has been a change in the distribution of paediatric cancers in SSA. Risk factors associated with paediatric cancer and trends in paediatric cancer in relation to HIV will also be examined. In extracting the relevant information, the inclusion and exclusion criteria and research question will be put into consideration. The tool will be piloted by two independent reviewers to check for consistency. Ten included studies will be selected at random for piloting the form and the form modified if necessary prior to its final use.

**Table 2:** Data extraction form

Study details	
Author and date	
Objectives/ research questions	
Study population	
Sample size	
Sex	
Study setting (country of origin of included studies)	
Interventions	
Study outcome/findings	
Relevant outcomes/findings assessed	

## Collating, summarising and reporting the results

After charting the data, a descriptive overview of the studies will be presented in a narrative format. An account of the number of studies, research methods used, gender percentage, types and distribution of paediatric cancers and outcomes will be described in this narrative. The analysis will be mainly based on mapping the country-specific outcomes emerging from the studies and a numerical summary of these outcomes will be conducted. A table for the latter will be produced. In analysing the outcomes, a comparison of the malignancies amongst the SSA countries will be made. A geographical map of the individual countries reported in the studies and charts showing the types of cancers will also be produced and presented in the results section. We will also analyse the meanings of the findings in relation to the aim of the study and the implications of these for future research, policy and practice in SSA.

## Discussion

The objective of this scoping review is to map existing literature for evidence on the distribution of paediatric cancers, epidemiological trends in paediatric cancers and trends in paediatric cancer in relation to HIV in SSA. A map on the distribution of childhood cancers in the SSA region will give a reflection on the disease burden, assessment and identification of the aetiology and risk factors. There is a high demand of evidence synthesis studies that are applicable to the decision-makers’ local context, to help improve health systems’ performance and subsequently improve health outcomes [13].

This study will be the first to map existing literature for evidence on the distribution of childhood cancers in the SSA region. A systematic review was conducted on the epidemiology of childhood hematologic cancers in Africa [14]. The study provided a comparison of pooled incidence rates amongst the different African regions from different studies and suggested an increase in haematological cancers in the African continent [14]. However, the report was only on haematological cancers and excluded other solid tumours.

The data reported by the WHO/IARC on the burden of paediatric cancers is the most extensive so far [2, 15]. However, these figures have been disputed as being an under-estimation of the actual distribution in SSA [1]. Registration methods differ worldwide; furthermore, the availability of data sources, classification standards and data coverage differ depending on the socio-economic status in the world regions, thus influencing the incidence rates. Now more than ever, there is a need for further research and on the spectrum and distribution of paediatric cancers in the SSA and other low-income countries region so as to provide such organisations with high quality and comparable data. The importance of data on cancer cannot be overemphasised, as it provides an insight on the actions that are needed and groups in a population that should be prioritised [16]. Accurate epidemiological data on paediatric cancers will enable mobilisation of sufficient resources for proper screening, prevention and treatment of these cancers. This review study will identify existing research gaps for future research to influence policy implementation and to improve the availability of diagnosis and treatment of paediatric cancers in SSA.

Only studies focusing on childhood malignancies will be included. The age range for this study is 0–14 years as it is the commonly used age range in most paediatric disease studies. To minimise bias, there will be no language restriction in selecting the studies. Since the study is aimed at mapping the distribution of paediatric cancers, quantitative studies will be included and qualitative studies will be excluded.

### Limitations of the study

The limitation to this study is that the study findings may be broad due to the broad nature of the research question and the authors might require additional steps to synthesise and come up with relevant conclusions. Also due to the broad focus of this scoping review, selection of databases and use of search terms, some studies might be missed. It can be argued that since scoping reviews emphasise more on the comprehensiveness of literature rather than the quality of evidence the quality appraisal step does not apply. However, gaps in literature related to low-quality study designs may not be ascertained.

## Conclusion

It is anticipated that the results of this study will give a reflection of paediatric cancer burden, assessment and identification of risk factors associated with paediatric cancer in the SSA region. Furthermore evidence synthesized from this study will help researchers and local decision makers in improving the perfomance of health systems, thus improving treatment and survival of children living with cancer.

## Data Availability

All data generated or analysed during this study will be included in the published scoping review article.

## Appendix

**Table 3:** Pilot search results from PubMed database

Date of search	Search engine used	Keyword search	Number of publications retrieved
19 Nov. 2018	PubMed	(((((((“Childhood”[Journal] OR “childhood”[All Fields]) AND (“neoplasms”[MeSH Terms] OR “neoplasms”[All Fields] OR “cancer”[All Fields])) OR ((“pediatrics”[MeSH Terms] OR “pediatrics”[All Fields] OR “paediatric”[All Fields]) AND (“neoplasms”[MeSH Terms] OR “neoplasms”[All Fields] OR “cancer”[All Fields]))) AND (“risk factors”[MeSH Terms] OR (“risk”[All Fields] AND “factors”[All Fields]) OR “risk factors”[All Fields])) AND (“epidemiology”[Subheading] OR “epidemiology”[All Fields] OR “prevalence”[All Fields] OR “prevalence”[MeSH Terms])) OR (“mortality”[Subheading] OR “mortality”[All Fields] OR “mortality”[MeSH Terms])) AND (“epidemiology”[Subheading] OR “epidemiology”[All Fields] OR “morbidity”[All Fields] OR “morbidity”[MeSH Terms])) AND trend[All Fields]	21,212

## Abbreviations

IARC: International Agency for Research in Cancer
MeSH: Medical Subject Headings
MMAT: Mixed Method Appraisal Tool
PEO: Population, Exposure and Outcome
PRISMA: Preferred Reporting Items for Systematic Reviews and Meta-Analyses
SSA: Sub-Saharan Africa
WHO: World Health Organization
